# Supplementary material for: A Comprehensive Prognostic Analysis of Tumor-Related Blood Group Antigens in Pan-Cancers Suggests That SEMA7A as a Novel Biomarker in Kidney Renal Clear Cell Carcinoma
Source: Int J Mol Sci. 2022 Aug 8;23(15):8799. doi: 10.3390/ijms23158799 (PMC9369114; doi:10.3390/ijms23158799)
Supplement: Supplementary file 1 [file ijms-23-08799-s001.zip › ijms-1816901-supplementary.pdf]

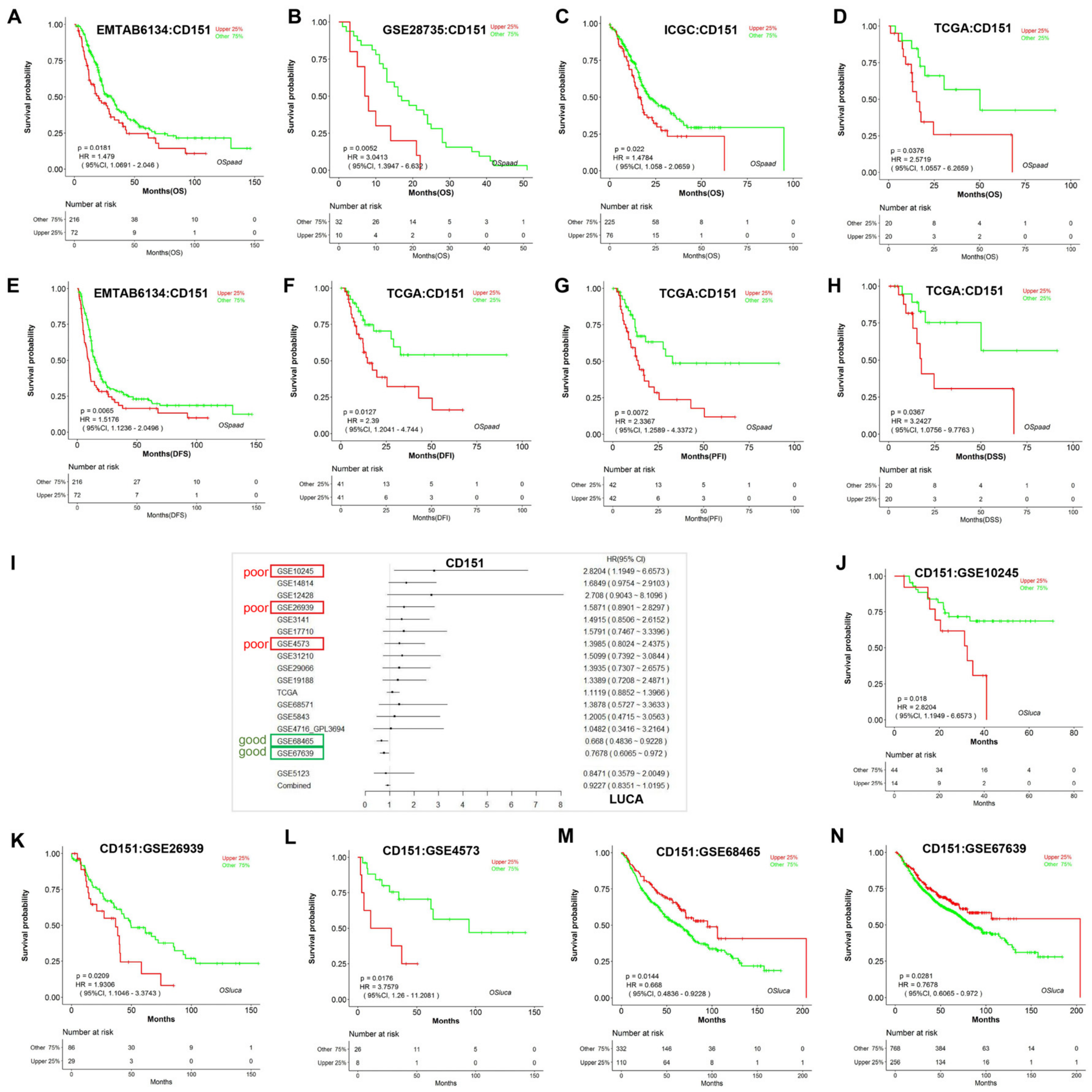

Figure S1. Kaplan-Meier survival curve of cancers with high and low CD151 expression analyzed by the LOGpc database.

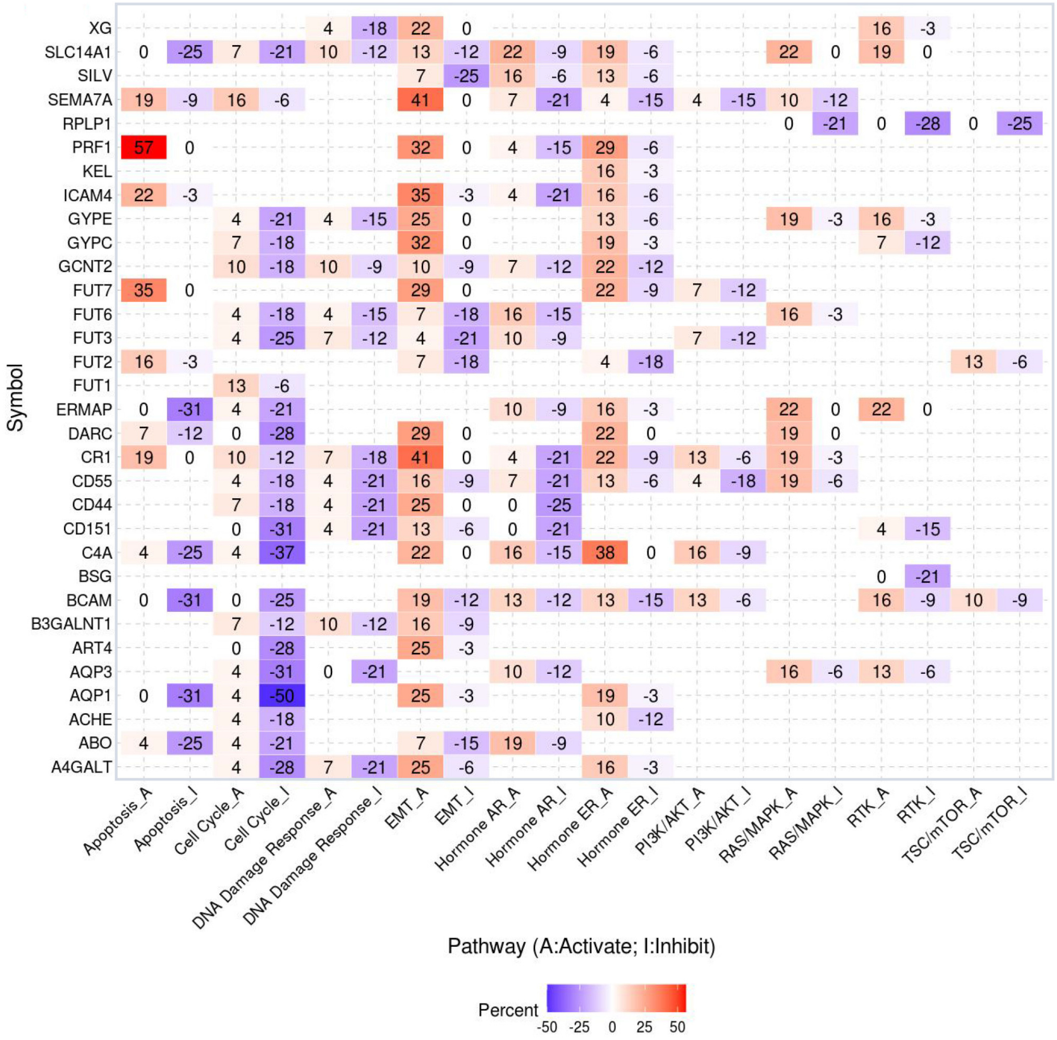

Figure S2. Summary of the degree of antigen gene activation or inhibition in the classical cancer-pathways.

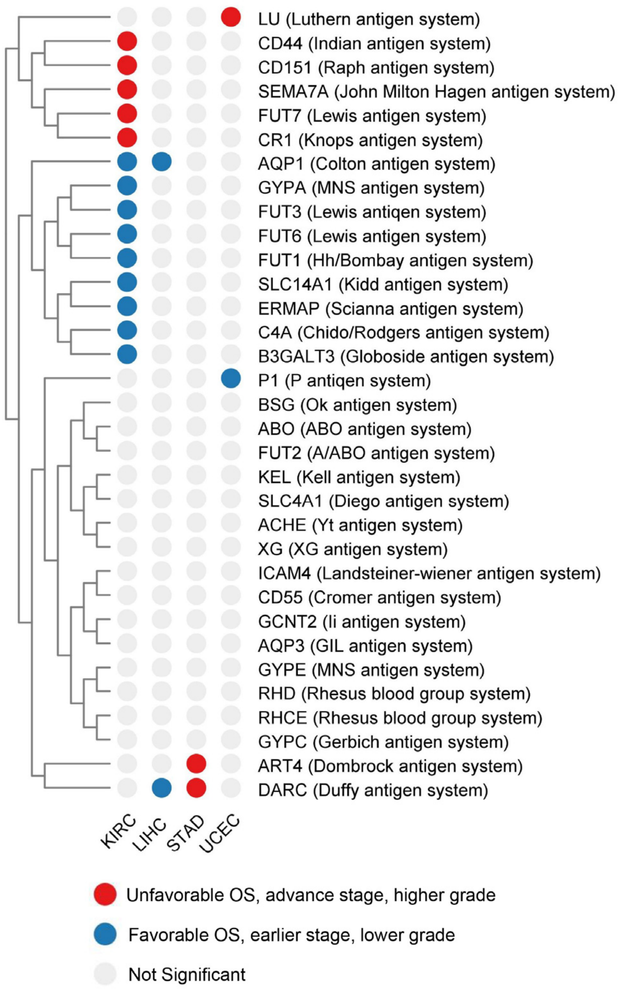

Figure S3. The association of antigen gene expression with OS, stage and grade.
